# *Staphylococcus capitis* isolated from prosthetic joint infections

**DOI:** 10.1007/s10096-016-2777-7

**Published:** 2016-09-29

**Authors:** S. Tevell, B. Hellmark, Å. Nilsdotter-Augustinsson, B. Söderquist

**Affiliations:** 1 Department of Infectious Diseases, Karlstad Hospital, Karlstad, Sweden; 2 School of Medical Sciences, Faculty of Medicine and Health, Örebro University, Örebro, Sweden; 3 Department of Laboratory Medicine, Faculty of Medicine and Health, Örebro University, Örebro, Sweden; 4 Department of Infectious Diseases and Department of Clinical and Experimental Medicine, Linköping University, Linköping, Sweden

## Abstract

Further knowledge about the clinical and microbiological characteristics of prosthetic joint infections (PJIs) caused by different coagulase-negative staphylococci (CoNS) may facilitate interpretation of microbiological findings and improve treatment algorithms. *Staphylococcus capitis* is a CoNS with documented potential for both human disease and nosocomial spread. As data on orthopaedic infections are scarce, our aim was to describe the clinical and microbiological characteristics of PJIs caused by *S. capitis*. This retrospective cohort study included three centres and 21 patients with significant growth of *S. capitis* during revision surgery for PJI between 2005 and 2014. Clinical data were extracted and further microbiological characterisation of the *S. capitis* isolates was performed. Multidrug-resistant (≥3 antibiotic groups) *S. capitis* was detected in 28.6 % of isolates, methicillin resistance in 38.1 % and fluoroquinolone resistance in 14.3 %; no isolates were rifampin-resistant. Heterogeneous glycopeptide-intermediate resistance was detected in 38.1 %. Biofilm-forming ability was common. All episodes were either early post-interventional or chronic, and there were no haematogenous infections. Ten patients experienced monomicrobial infections. Among patients available for evaluation, 86 % of chronic infections and 70 % of early post-interventional infections achieved clinical cure; 90 % of monomicrobial infections remained infection-free. Genetic fingerprinting with repetitive sequence-based polymerase chain reaction (rep-PCR; DiversiLab®) displayed clustering of isolates, suggesting that nosocomial spread might be present. *Staphylococcus capitis* has the potential to cause PJIs, with infection most likely being contracted during surgery or in the early postoperative period. As *S. capitis* might be an emerging nosocomial pathogen, surveillance of the prevalence of PJIs caused by *S. capitis* could be recommended.

## Introduction

*Staphylococcus* spp. are the most common cause of prosthetic joint infections (PJIs). In different series, coagulase-negative staphylococci (CoNS) occur in 13–41 % of infections [1]. Previous routine practice has usually been to identify *S. epidermidis* and *S. lugdunensis* to the species level but report all others to the clinician as CoNS. This might pose a particular problem for PJIs, as case definitions include growth of low-virulence pathogens (i.e. CoNS) in ≥2 perioperative tissue samples [2, 3]. Thus, it might make a difference if two isolated CoNS belong to the same species (indicating infection) or two different species (indicating contamination). Differences in antibiotic resistance patterns are used in the attempt to distinguish between different CoNS species [4], but no data support this practice in diagnosing PJIs.

However, the implementation of new methods, such as matrix-assisted laser desorption/ionisation time-of-flight mass spectrometry (MALDI-TOF MS) or real-time broad-range polymerase chain reaction (PCR), has simplified the identification of CoNS to the species level [5, 6]. This might contribute to better understanding of the epidemiology and pathogenicity of these bacteria in PJIs, as the CoNS act as both pathogens and commensals [7].

*Staphylococcus capitis* is a CoNS mainly distributed on the head (primarily ears and forehead), arms and occasionally legs. A previous study showed that 20 % of individuals maintained persistent populations on the head and arms over 1 year [8].

*Staphylococcus capitis* has two subspecies: subsp. *urealyticus* and subsp. *capitis*. The former, but not the latter, has been shown to increase its range of habitats outside the head during antibiotic therapy.

Both subspecies are classified as human pathogens [9]. Case reports indicate that *S. capitis* plays a role in continuous ambulatory peritoneal dialysis peritonitis [10], prosthetic-valve endocarditis [11], pacemaker endocarditis [12], meningitis [13], acetabulum osteomyelitis [14] and spondylodiscitis [15].

It is also a well-recognised pathogen in neonatal sepsis, where its capacity for clonal nosocomial spread has been verified by genetic fingerprinting [16, 17]. Investigation of nine *S. capitis* isolates from blood cultures in neonatal intensive care units (NICUs) showed that all of them exhibited vancomycin-heteroresistant subpopulations [18].

*Staphylococcus capitis* has only rarely been identified to the species level in routine clinical practice, even when obtained from PJIs. However, recent reports focusing on further subtyping of bone and joint infection isolates have identified *S. capitis* as a causative organism [6, 7, 19, 20], though its role as a pathogen has been debated. *Staphylococcus capitis* has also been shown to be the predominant CoNS in laminar air flow during prosthetic joint surgery [21]. Therefore, the aim of this retrospective cohort study was to describe the clinical and microbiological characteristics of PJIs caused by *S. capitis*.

## Materials and methods

### Study population

The study population was recruited from three centres in central Sweden, representing approximately one million inhabitants; the county councils of Värmland (Karlstad Central Hospital), Östergötland (Linköping University Hospital) and Örebro (Örebro University Hospital). All patients were identified by the laboratory information systems and the presence of CoNS in perioperative tissue biopsies retrieved from revision surgery for hip or knee PJI. As there are no private laboratories processing microbiological samples in these regions, this process will have identified all episodes of PJIs resulting in surgery with tissue biopsies for culture.

PJI was defined according to the criteria of the Infectious Diseases Society of America (IDSA) [2]. A total of 21 patients diagnosed between 2005 and 2014 were followed from the PJI diagnosis until the end of the study, treatment failure or death. Clinical cure was defined as ≥24 months without recurrence of infection after the end of treatment. In cases where clinical cure was presumed but follow-up was ≤24 months, the result was defined as non-available (n/a).

The study was approved by the Regional Ethical Review Board of Uppsala (ref: 2014/418).

### Bacterial isolates

CoNS isolated in ≥2 perioperative tissue samples obtained from revision surgery for hip or knee arthroplasties due to PJI were included for further analysis. If there were not at least two CoNS isolates with similar colony morphology and antimicrobial resistance pattern, the patient was excluded. MALDI-TOF MS was performed for species identification using a Microflex LT (Bruker Daltonik GmbH, Bremen, Germany) and MALDI Biotyper software version 3.1 (Bruker Daltonik), with a required score threshold of ≥2.0. Further verification was performed by *rpoB* sequencing of four isolates (#R1, #R2, #12, #15) representing different clusters in the DiversiLab dendrogram. All staphylococci other than *S. capitis* were excluded. Polymicrobial infections, whether caused by staphylococci or other pathogens, were included as long as *S. capitis* was found in at least two tissue cultures. To discriminate between subsp. *capitis* and *urealyticus*, urease activity and maltose fermentation tests were performed [22], using both tube test and ID32 STAPH (bioMérieux, Marcy l’Etoile, France).

All isolates were stored at −70 °C at the clinical laboratory in each county, then subcultured at 35 °C overnight on Columbia II agar (BD Diagnostic Systems, Sparks, MD, USA) supplemented with 6 % horse blood (SVA, Uppsala, Sweden).

Reference isolates CCUG 55892 (*S. capitis* subsp. *urealyticus*) and CCUG 35173 (*S. capitis* subsp. *capitis*) were used.

### Antibiotic susceptibility testing

Standard antibiotic susceptibility testing by the disc diffusion test was performed for cefoxitin (30 μg), fusidic acid (10 μg), erythromycin (15 μg), clindamycin (2 μg), trimethoprim/sulphamethoxazole (25 μg), gentamicin (10 μg), norfloxacin (10 μg), ciprofloxacin (5 μg), rifampin (5 μg) and vancomycin (5 μg) (all antibiotic discs from Oxoid, Basingstoke, Hampshire, England), with a 0.5 McFarland bacterial suspension in 0.85 % NaCl on Mueller–Hinton agar (Oxoid). After 16–20 h of incubation at 36 °C, the zone diameters were measured and each isolate was evaluated according to European Committee on Antimicrobial Susceptibility Testing (EUCAST) breakpoints (http://www.eucast.org, accessed 2015-03-20).

Standard minimum inhibitory concentration (MIC) determination with the Etest (bioMérieux) was performed for vancomycin and teicoplanin on Mueller–Hinton agar (Oxoid) with a bacterial suspension adjusted to 0.5 McFarland in 0.85 % NaCl. The results were determined after 24 h of incubation at 36 °C, and, again, each isolate was evaluated according to EUCAST breakpoints.

Isolates resistant to ≥3 antibiotic groups tested were considered multidrug-resistant (MDR).

### Screening for heterogeneous glycopeptide-intermediate *S. capitis*

The detection of heterogeneous glycopeptide-intermediate *S. capitis* (hGISC) was performed using the VAN4 method [17, 18, 23]. Briefly, an overnight blood agar plate culture was suspended in 0.9 % NaCl and adjusted to 0.5 McFarland turbidity. Four droplets (10 mL) of the suspension were dropped onto a BHI agar plate (BD Diagnostic Systems) containing 16 g/L pancreatic digest of casein (BD Diagnostic Systems) and 4 mg/mL vancomycin. After 48 h of incubation at 35 °C, the colonies in each droplet were counted. If ≥1 droplet had ≥2 colonies, the strain was considered to be heteroresistant to vancomycin. In addition, the macromethod Etest (MME) and glycopeptide resistance detection (GRD) Etest were performed as previously described [24].

### Biofilm

For the detection of biofilm production, both microtitre plate assay (MTP) and Congo red agar assay (CRA) were used, as previously described, with the modification of 48 h of incubation at 36 °C for the CRA assay instead of 24 h [25–27]. *Staphylococcus epidermidis* RP62A was used as the positive control and *S. epidermidis* ATCC 12228 as the negative control.

### Repetitive sequence-based PCR (DiversiLab®)

The semi-automatic repetitive sequence-based PCR (rep-PCR) microbial genotyping system (DiversiLab®) was used to detect the genomic fingerprints of each *S. capitis* isolate as previously described [28], with the exception of using the DiversiLab® Staphylococcus fingerprinting kit (bioMérieux, Marcy l’Etoile, France), following the manufacturer’s instructions.

### Statistics

Version 18 of the SPSS software package (IBM SPSS, Chicago, IL, USA) was used for data handling.

## Results

### Species identification

No isolates were identified as *S. capitis* subsp. *capitis*, 13 isolates (61.9 %) were identified as *S. capitis* subsp. *urealyticus* and 8 isolates (38.6 %) were untypeable; that is, they were urease-negative and maltose-positive despite using two different methods. There were no divergent results between the two methods among these 21 analyses. However, the reference isolate CCUG 55892 (*S. capitis* subsp *urealyticus*) was not identified as subsp. *urealyticus* (urease-negative/maltose-positive in tube fermentation, urease-negative/maltose-negative using ID32 STAPH).

### Antimicrobial susceptibility testing

Resistance to methicillin was found in 38.1 % of isolates, to erythromycin in 33.3 %, to clindamycin in 23.8 %, to gentamicin in 38.1 %, to ciprofloxacin in 13.6 %, to norfloxacin in 14.3 % and to fusidic acid in 4.8 %. No isolates were resistant to rifampin, linezolid, trimethoprim/sulphamethoxazole, teicoplanin or vancomycin. The mean vancomycin MIC was 1.25 mg/L (range 0.75–1.25 mg/L) and the mean teicoplanin MIC was 0.5 mg/L (range 0.094–1.0 mg/L). No isolates were identified as hGISC under either MME or GRD Etests, while 38.1 % of isolates were hGISC according to the VAN4 method. A total of 28.6 % of isolates were MDR and among those 83.3 % were hGISC.

### Biofilm formation

**Fig. 1:**
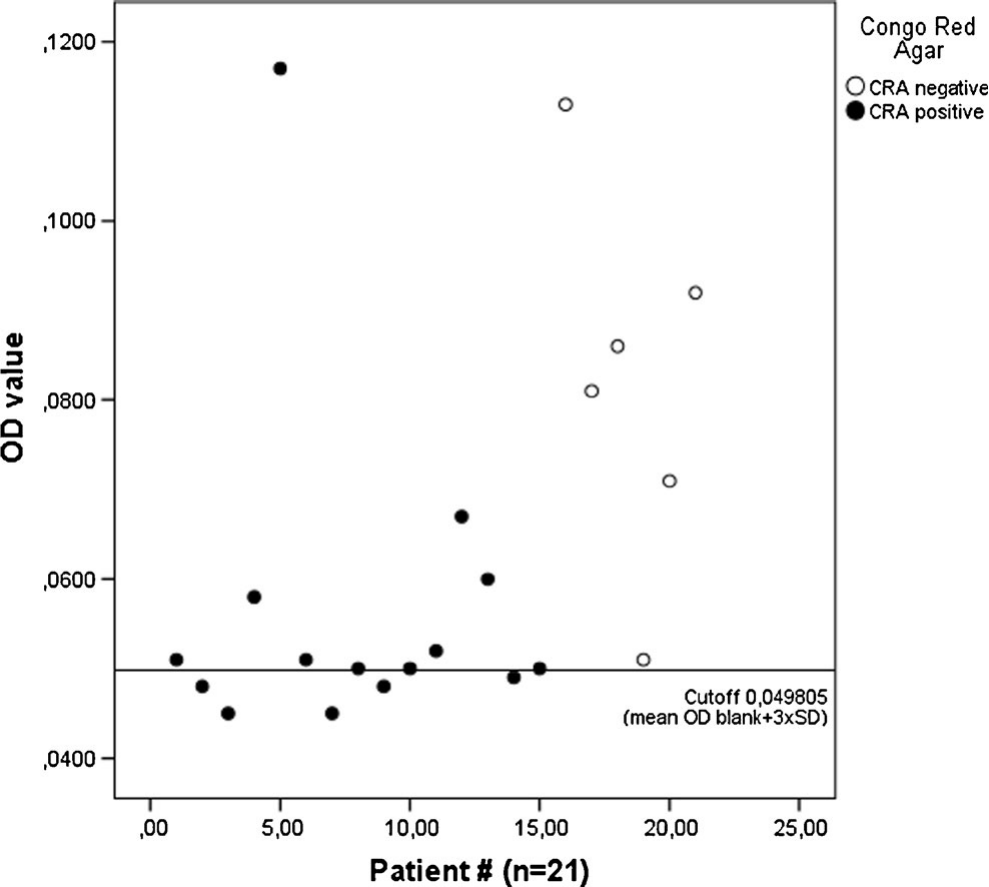
*Staphylococcus capitis* (*n* = 21) isolated from prosthetic joint infections (PJIs). The Congo red agar (CRA) results are compared to optical density (OD) values in the microtitre plate assay (MTP) in relation to the MTP cut-off; the CRA-negative isolates display a wide range of OD, while the majority of CRA-positive isolates cluster close to the cut-off

The two methods were not consistent in the detection of biofilm formation in *S. capitis* (Fig. 1). CRA detected biofilm formation in 71.4 % of isolates, while the MTP assay was positive, although displaying low values of optical density (OD), in 76.2 %. All isolates were positive in at least one of the two methods, and 47.6 % of isolates were positive in both methods.

### Molecular epidemiology of clinical *S. capitis* isolates from PJIs in a 10-year period

**Fig. 2:**
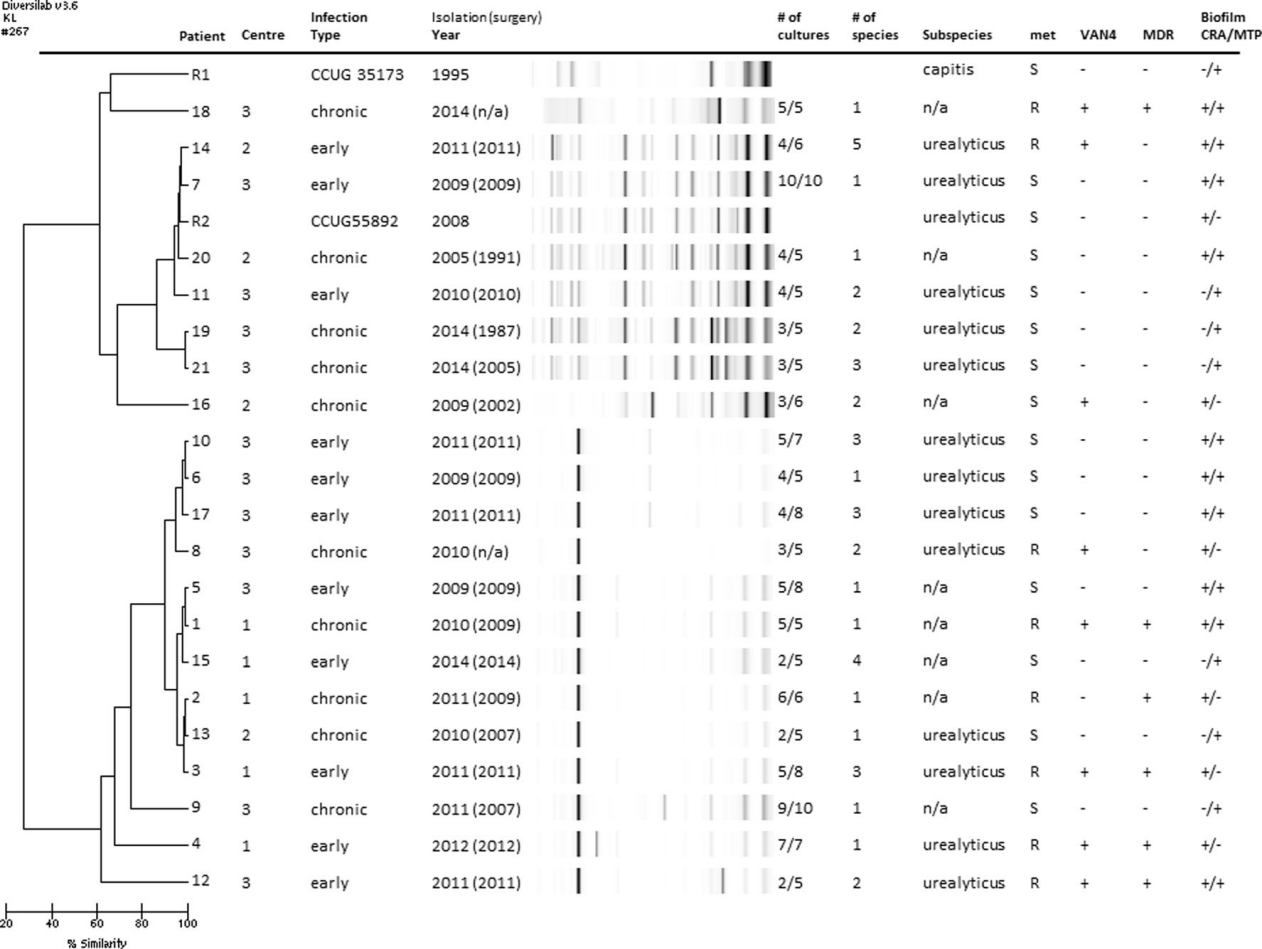
Dendrogram created with DiversiLab® including information about centre (centre 1: *n* = 5, centre 2: *n* = 4, centre 3: *n* = 12), infection type, year of isolation, i.e. diagnosis of PJI (year of primary surgery), number of tissue cultures displaying growth of *S. capitis*/total number of cultures, total number of pathogens in significant amount (≥2 tissue cultures or ≥1 if highly pathogenic bacteria such as *S. aureus*), subspecies, methicillin sensitivity (met), heterogeneous glycopeptide-intermediate *S. capitis* (using the VAN4 screening method), multidrug-resistance (MDR) and biofilm-forming ability (CRA = Congo red agar, MTP = microtitre plate assay)

Using the DiversiLab® software, a dendrogram (Fig. 2) was created to assess similarities between the isolates. The dendrogram showed two distinct clusters with highly divergent gel patterns. Both reference isolates were located in the upper cluster, even though they belonged to different subspecies. Isolates from patients 19 and 21 were both isolated on the same day in the same centre. Four isolates from centre 3 originating between 2009 and 2011 (6, 8, 10 and 17) were clustered together in the lower cluster, as were four isolates from centre 1 originating between 2009 and 2014 (1, 2, 3 and 15).

**Fig. 3:**
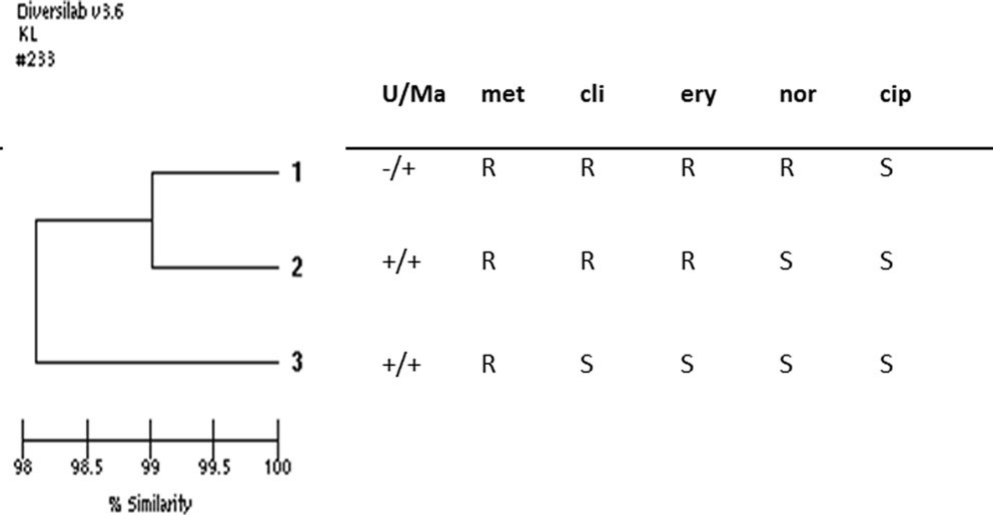
Dendrogram created with DiversiLab® combined with the results of biochemical analyses from three simultaneously collected isolates from patient 1 during surgery. This infection was monomicrobial. U/Ma, urease/maltose; met, methicillin; cli, clindamycin; ery, erythromycin; nor, norfloxacin; cip, ciprofloxacin

Figure 3 shows a dendrogram illustrating the similarity between three perioperative isolates obtained simultaneously from patient 1, with the results of biochemical analysis and antimicrobial susceptibility testing added. The dendrogram displays close clustering between the three isolates, although the biochemical phenotype and antimicrobial susceptibility patterns differ.

### Clinical data

The patient characteristics are outlined in Table 1. Ten of the infections were monomicrobial and another three contained other CoNS species in addition to *S. capitis*. Eleven infections were early post-interventional (infection ≤1 month after surgery), while the remaining cases were chronic infections or were diagnosed during surgery for suspected aseptic loosening. There were no episodes of acute haematogenous infection.

**Table 1:** Characteristics of 21 patients diagnosed with prosthetic joint infections (PJIs) with significant growth of *Staphylococcus capitis*

Baseline data	*n* = 21
Male sex	15 (71 %)
Age (years)	65.8 (24–87)
Rheumatic disorder	2 (10 %)
Diabetes mellitus	1 (5 %)
Monomicrobial infection	10 (48 %)
Joint affected	
Hip	13 (62 %)
Knee	8 (38 %)
Antibiotics postoperative (final)	*n* = 21
Vancomycin	11 (52 %)
Beta-lactam	4 (19 %)
Clindamycin	3 (14 %)
None	2 (10 %)
Daptomycin	1 (5 %)
Antibiotics follow-up (final)	*n* = 21
Rifampin combination	10 (48 %)
Rifampin/fluoroquinolone	4 (19 %)
Rifampin/fusidic acid	4 (19 %)
Rifampin/clindamycin	1 (5 %)
Rifampin/linezolid	1 (5 %)
Surgery	*n* = 21
DAIR	11 (52 %)
Symptom->DAIR ≤21 days	9 (82 %)
One-stage exchange	3 (14 %)
Two-stage with spacer	5 (24 %)
Two-stage without spacer	2 (10 %)

*DAIR* debridement, antibiotics and implant retention

Wound healing disturbances were common in the early post-interventional infections (Table 2), regardless of whether the infection was mono- or polymicrobial. Pain was the most common symptom among patients with chronic infections. The patients with monomicrobial early post-interventional infections expressed the highest levels of C-reactive protein and the patients with monomicrobial chronic infections expressed the lowest.

**Table 2:** Clinical symptoms, signs, outcome and type of infection among 21 patients with PJIs caused by *S. capitis*

		Wound healing disturbance	Sinus tract	Pain	Fever	Cemented prosthesis	CRP range	CRP median	CRP mean	Type of revision surgery	Clinical cure	Remaining infection	Deceased	n/a
Early post-interventional infection														
Monomicrobial (*n* = 4)	Yes	3 (75 %)	0 (0 %)	2 (50 %)	1 (25 %)	4 (100 %)	10–406	55	131.5	DAIR	4	0	0	0
	No	1 (25 %)	4 (100 %)	2 (50 %)	3 (75 %)	0 (0 %)				Two-stage	0	0	0	0
Polymicrobial (*n* = 7)	Yes	5 (71 %)	0 (0 %)	3 (43 %)	3 (43 %)	6 (86 %)	1–186	33	58.8	DAIR	3	2	0	1
	No	2 (29 %)	7 (100 %)	4 (57 %)	4 (57 %)	1 (14 %)				Two-stage	0	1	0	0
											*7/10 (70 %)*			
Chronic infection														
Monomicrobial (*n* = 6)	Yes	1 (17 %)	1 (17 %)	5 (83 %)	0 (0 %)	4 (67 %)	4–16	7.5	8.8	One-stage	2	0	0	0
	No	1 (17 %)	5 (83 %)	1 (17 %)	6 (100 %)	2 (33 %)				Two-stage	2	0	1	0
										Cup revision	1	0	0	0
Polymicrobial (*n* = 4)	Yes	0 (0 %)	0 (0 %)	4 (100 %)	0 (0 %)	3 (75 %)	2–48	9	19.7	One-stage	0	0	0	1
	No	1 (25 %)	4 (100 %)	0 (0 %)	4 (100 %)	1 (25 %)				Two-stage	1	0	0	2^a^
											*6/7 (85.7 %)*			

*CRP* C-reactive protein (mg/L); *DAIR* debridement, antibiotics and implant retention

^a^One of these two patients is expected to have achieved clinical cure, but follow-up was <24 months at the time of writing

All chronic infections were treated with prosthesis exchange surgery (six two-stage and three one-stage exchange), except one episode diagnosed during cup revision. Clinical cure was achieved in six of the seven assessable cases (85.7 %). Of the early post-interventional infections, ten were treated with debridement, antibiotics and implant retention (DAIR) and one with two-stage exchange; seven of the ten assessable cases (70.0 %) achieved clinical cure. All ten patients with monomicrobial *S. capitis* infections, except one who unexpectedly died within the first week after surgery, achieved clinical cure.

## Discussion

This cohort study of PJIs caused by *S. capitis* combines clinical data with microbiological characterisation of the isolates. We present 21 cases, including ten monomicrobial infections, where *S. capitis* has been isolated in ≥2 tissue samples obtained during revision surgery for PJIs, thus fulfilling the criteria for PJI. None of the cases were acute haematogenous, suggesting that the main route of transmission for *S. capitis* is perioperative or early in the postoperative phase. The finding that *S. capitis* is prevalent in the air of operation rooms may further support this hypothesis [21].

To our knowledge, no previous data regarding rep-PCR in the setting of *S. capitis* are available, as most work is performed on *S. aureus*. However, in a recent publication on *S. caprae* in bone and joint infections [29], rep-PCR was compared to pulsed-field gel electrophoresis (PFGE), with the conclusion that it may be a good screening method. In our analysis, there were three interesting clusters in the dendrogram obtained with rep-PCR that warrant further discussion. The first and second clusters included four isolates each from two different centres (6, 8, 10 and 17 from centre 3; 1, 2, 3 and 15 from centre 1). Some of these episodes were temporally close, with only 4 months between the primary surgery in patients 11 and 13, 5 months between patients 5 and 11, and 8 months between patients 16 and 18. Also, four of the five isolates from centre 1 exhibited the MDR phenotype. This, combined with knowledge from observations from NICUs [16, 17] and bloodstream isolates [30], is suggestive of nosocomial clonal spread of *S. capitis* causing PJIs.

The third cluster consisted of patients 19 and 21, whose samples were isolated at the same centre on the same day by the same surgeon during revision surgery for suspected chronic infection. Multiple CoNS were obtained in cultures from patient 19, with growth of *S. epidermidis* with two different antibiotic susceptibility patterns in all five samples, *S. capitis* in three samples and *S. hominis* in one. No antibiotic treatment was given, and the patient has not requested further intervention. Multiple staphylococci were also obtained in patient 21: *S. capitis* in three of five samples, *S. epidermidis* in three and *S. aureus* in one. However, during revision surgery 6 weeks later, no bacteria were found in the tissue samples. As primary surgery in these two patients was performed 8 years apart, contamination in the operating theatre seems plausible, even though both these cases fulfil the criteria for infection. This highlights the importance of adequate handling of samples throughout the entire chain, from the operating theatre to the microbiological laboratory.

Thus, some of these positive cultures might indicate contamination, even though they fulfilled the criteria for PJI. Figure 3 further demonstrates the complexity in diagnosing PJIs caused by *S. capitis*. This patient presented a sinus tract 17 months after primary knee arthroplasty. Without subtyping, the clinician would probably have interpreted the finding of three CoNS with different antimicrobial susceptibility patterns as a contamination. Subtyping demonstrated that all isolates were *S. capitis*, but biochemical tests differed regarding maltose fermentation and urease activity, still suggesting polyclonal contamination. However, rep-PCR analysis, which is not used in routine clinical practice, demonstrated >98 % similarity between the isolates, indicating infection despite the phenotypic divergences.

All typeable *S. capitis* consisted of subsp. *urealyticus*, which has previously been shown to be more likely to express biofilm in vitro, and also expresses more extensive antibiotic resistance compared to subsp. *capitis* [31]. However, we discovered difficulties in further distinguishing between these two subspecies using maltose/urease biochemical tests. By using two different methods, we tried to eliminate the possibility that this phenomenon might be solely methodological. Other potential explanations for this include differences in phenotypic expression or, as has been proposed earlier [19], the presence of a novel subspecies of *S. capitis*.

Regarding the assays for the detection of biofilm formation, the MTP assay generally yielded very low levels of OD, giving a poor separation when calculating the cut-off as the mean of the negative control +3SD. Even if the importance of the *ica* locus in biofilm production of *S. capitis* is not clearly defined [31, 32], future comparison of the genomes of these isolates might provide further insights regarding both biofilm formation and subtype.

Outcomes were generally favourable, especially in monomicrobial infection. There was less multidrug resistance as well as a lower presence of glycopeptide-heteroresistant subpopulations among these isolates compared with *S. epidermidis* isolated from PJIs from the same region [24]. Regarding the detection of glycopeptide-heteroresistant subpopulations, the time-consuming population analysis profile/area under the curve (PAP-AUC) is regarded to be the gold standard, while reliable screening methods are lacking. MME and GRD Etests are methods validated for *S. aureus*, although a few reports are available for *S. epidermidis* as well [24, 33]. To our knowledge, these methods have not been applied to *S. capitis*, while VAN4 has been compared to PAP-AUC and standard Etest in a limited number of isolates [18]. When analysing the PJI isolates presented in this paper, no heteroresistant subpopulations were detected using MME and GRD Etests, while 38.1 % of isolates were hGISC according to VAN4. Among the MDR isolates, the prevalence was even higher. Since heteroresistant subpopulations might be difficult to detect by screening methods, these results suggest that MME and GRD Etests might be insufficient for use in the setting of *S. capitis*.

In conclusion, *S. capitis* has the potential to cause PJIs, both on its own and as part of polymicrobial infections. Antibiotic susceptibility patterns are generally more favourable compared with *S. epidermidis* isolated from PJIs. It is most likely that PJI caused by *S. capitis* is contracted during surgery or in the early postoperative period. Furthermore, since fingerprinting with rep-PCR displayed clustering within and between centres, surveillance of the prevalence of PJIs caused by *S. capitis* could be recommended in order to allow swift action if nosocomial spread occurs.
